# The David Sign, Revisited

**DOI:** 10.1016/j.ajmo.2023.100061

**Published:** 2023-10-30

**Authors:** Daniel M. Gelfman

**Affiliations:** Division of Clinical Affairs, Marian University College of Osteopathic Medicine, Indianapolis, Ind, United States of America

**Keywords:** The David Sign, Michelangelo, Jugular venous distention, External jugular vein, Anterior jugular vein

## Abstract

This commentary discusses a new, previously unrecognized, undocumented, anatomic finding concerning the jugular venous distention present on one of the world’s most famous statues, Michelangelo’s David. This finding is provocative as it involves of one of Michelangelo’s “messages” being demonstrated in The David, has clinical relevance, and, appears to reveal something about Michelangelo, himself.

*The David* (1504)*,* by Michelangelo (1475-1569), is one of the most famous and visited statues in the world. Giorgio Vasari (1511-1574), who is the first and most famous biographer of the artists of the Renaissance, wrote: “Without any doubt this figure has put in the shade every other statue, ancient or modern, Greek or Roman … To be sure, anyone who has seen Michelangelo's David has no need to see anything else by any other sculptor, living or dead.”^1^ Since going on display in Florence, Italy, it has been viewed and revered by millions. Much has been written about *The David*, but not until recently (2019) was the presence of enigmatic external jugular venous distention (JVD) commented upon.

The article “The David Sign,” in conjunction with a response to a letter to the editor, discusses Michelangelo's depiction of “sustained” JVD in several of his sculptures, most notably *The David, Moses* (1515), and *Brutus* (1542) and interestingly not present in his *Pieta* (1499), as well as its presence in specific sculptures from antiquity (BCE 750 to 350 CE).^2^^,^^3^ Together these manuscripts discuss and offer explanations for this seemingly paradoxical finding, as sustained marked JVD in the upright position is not normal and suggests elevated intracardiac pressure or venous outflow obstruction. It is intriguing to try to understand what these artists were trying to convey, as they used their works of art, not words, to communicate their thoughts. Additionally, the presence or absence of JVD was not recognized as a sign of health or of disease until 1728 by Lancisi, and JVD was only portrayed in sculptures of healthy individuals.

The David Sign is thought to represent Michelangelo's thoughtful observation of intermittent JVD (which appears sustained when depicted in stone) related to forceful respiration, seen in excited individuals who are breathing heavily against a partially closed glottis as seen in grunting respiration, forceful speaking or singing, and exertion.^2^^,^^3^ In Michelangelo's sculpture of David, he is depicted just before his battle with Goliath, when he would have been energized and thus breathing forcefully. The JVD is not thought to represent underlying heart disease but does represent temporary compression of the heart from increased intrathoracic pressure and a temporary increase in right atrial pressure. The David Sign is most easily observed today in the exposed necks of singing actors in musicals and is easily differentiated from pathologic intermittent JVD related to each cardiac cycle, such as seen in severe tricuspid regurgitation or sustained elevation in JVD seen in heart failure. (For completeness, the left atrium is compressed at the same time as the right atrium, but so are the pulmonary capillaries, as they are intrathoracic, which prevents pulmonary congestion.)

I thought it might be of value to rephotograph the distended neck veins of *The David*, on a return visit to the Accademia Galleria in Florence, with greater magnification and from different angles, to see if there were additional details present that might be of interest. In reviewing those photographs, I notice a subtle additional finding, which, unlike the presence of external jugular vein distention, is extremely, if not impossibly, difficult to visualize from the ground without some form of telescopic magnification, as this sculpture is 17 feet tall. (The telescope had not been invented in 1504.) One should add that the initial plans for the statue were to place it quite high upon one of the Florentine Cathedral's elevated platforms.

What is visible with magnification is the subtle but clear presence of distention of the anterior jugular veins (Figures 1 and 2). Personally, I have never seen this vein displayed in sculpture, saying this as someone who has observed the necks of hundreds of sculptures. (Obviously one person's observations don't exclude the presence of this finding elsewhere, but it appears safe to state that this finding is at least extremely rare.) We know that Michelangelo did study superficial human anatomy and thus the presence of this vein with its connection to the external jugular vein was likely known to him. For clarification, the venous system at that time of Michelangelo was felt to deliver nutrients to the body and not to recirculate blood back to the heart as described by Harvey in 1628.^4^

**Figure 1:**
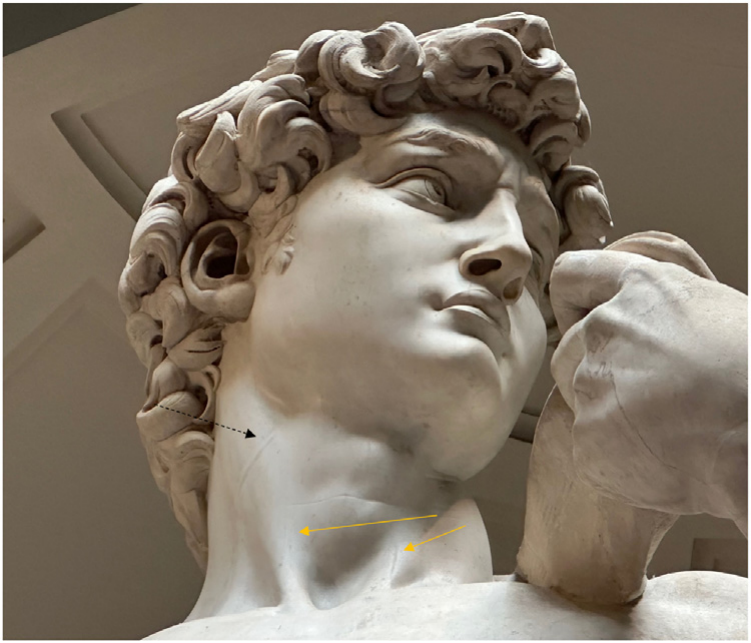
The David, demonstrating distention of the anterior jugular veins (gold solid arrows) and the external jugular vein (black line, dashed). Photo credit: Daniel M Gelfman, MD Image taken September 5, 2023.

**Figure 2:**
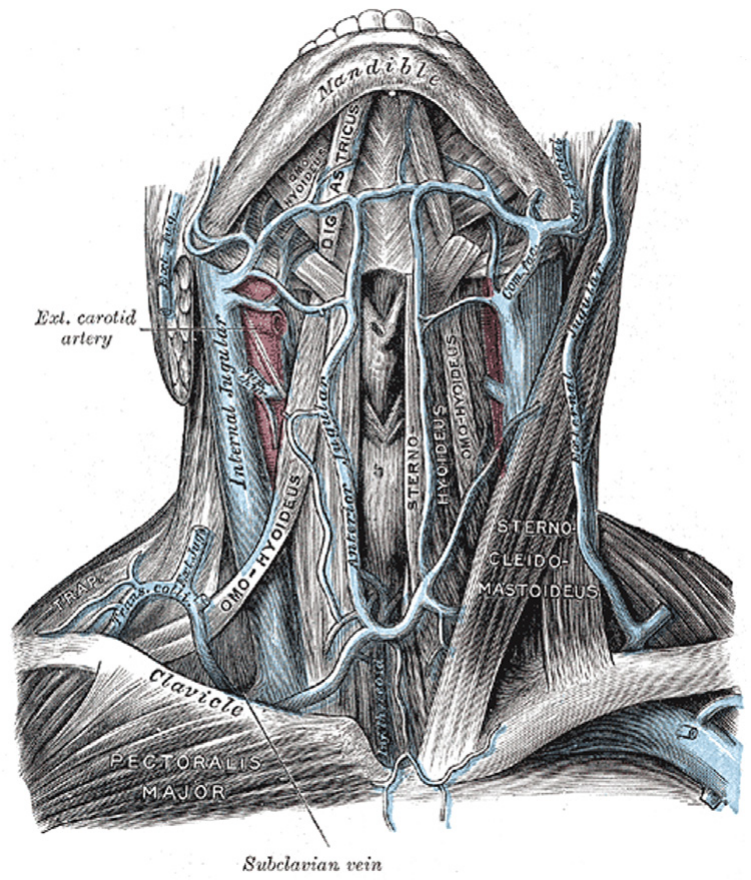
The location of the anterior and external jugular veins. Henry Grey. *Anatomy of the Human Body*. 20th ed., 1918. Under public domain. Downloaded from Wikipedia Commons October 17, 2023.

This attention to detail in sculpture, revealing the distention of the small anterior jugular veins in addition to more prominent external jugular vein appears unique and is provocative. It implies an understanding by the artist that the unusual distending force in the external jugular vein is also present in the anterior jugular veins. In addition, it suggests something about Michelangelo, himself. He must have known that his fine attention to detail and reality, while important to him to display, would go unrecognized for a very long time, possibly forever. Finally, it instructs us as medical practitioners, once again, about the need to closely observe the jugular veins, which can be visible in health and disease.

## Funding

None.

## Declaration of Competing Interest

None.
